# Effects of Vertically Heterogeneous Soil Salinity on Genetic Polymorphism and Productivity of the Widespread Halophyte *Bassia prostrata*

**DOI:** 10.3390/life13010056

**Published:** 2022-12-24

**Authors:** Elena Shuyskaya, Kristina Toderich, Alexander Kolesnikov, Maria Prokofieva, Marina Lebedeva

**Affiliations:** 1 K.A. Timiryazev Institute of Plant Physiology RAS, 35 Botanicheskaya St., 127276 Moscow, Russia; 2 International Platform for Dryland Research and Education, Tottori University, Tottori 680-0001, Japan; 3 Institute of Forest Science, Russian Academy of Sciences, 143030 Uspenskoe, Russia; 4 V.V. Dokuchaev Soil Science Institute, 7/2 Pyzhevsky per., 119017 Moscow, Russia

**Keywords:** plant performance, C_4_ species, heterozygosity, transient soil salinity, soil layers, desertification, arid regoins

## Abstract

Salinity is one of the environmental factors that affects both productivity and genetic diversity in plant species. Within the soil profile, salinity is a dynamic indicator and significantly changes with depth. The present study examined the effects of the vertical heterogeneity of soil salinity chemistry on the plant height, fresh and dry biomass accumulation, water content, level of genetic polymorphism, and observed and expected heterozygosity in seven populations of halophyte *Bassia prostrata* in natural habitats. Soil salinity ranged from slight (S_salts_ = 0.11–0.25%) to extreme (S_salts_ = 1.35–2.57%). The main contributors to salinity were Na^+^, Ca^2+^, and Mg^2+^. Multivariate analysis revealed that biomass accumulation is positively affected by moderate/high salinity in 20–60 cm soil layers, which may be associated with the salt required for the optimal growth of the halophyte *B. prostrata.* The formation of seed genetic diversity is negatively affected by slight/moderate salinity in the 0–40 cm layers. An increase in divalent ion content can reduce genetic diversity and increase the local adaptation of *B. prostrata* to magnesium–calcium sulfate salinity. The effect of the in-depth distribution of soil salinity on productivity and genetic diversity may be related to seasonal variables during biomass accumulation (summer) and seed formation (autumn).

## 1. Introduction

Salinity is a significant environmental problem that limits plant productivity, especially in arid and semiarid regions that cover approximately 40% of the globe. Semiarid regions are projected to become drier and more saline due to rising global temperatures [1,2,3]. Vegetation survival and productivity are primarily regulated by the water balance in soil, which affects the water balance and photosynthetic rate in plants [4]. Soils in drylands are usually heterogeneous in space and time due to the presence of biotic and abiotic elements. Spatiotemporal variations in soil salinity and water content are well documented [5]. Salinity amplifies the effects of soil drought on plants by creating additional osmotic pressure. Soil is considered saline when the salt content exceeds 3–5 g salt/L in the soil solution, when electrical conductivity (EC) exceeds 2–4 dS/m, or when the sum of salts exceeds 0.15–0.2%, creating osmotic pressure above 0.2 MPa, which significantly reduces the yield of the most crops [2,6]. Salinity reduces plant growth and prematurely ages mature leaves, which leads to a decrease in the functional leaf area. A decrease in plant biomass is also influenced by Na^+^ and Cl^−^ toxicity and the accompanying oxidative stress [2,7]. Halophytes are highly salt-tolerant plants but underutilized resources that occupy naturally saline soil environments in coastal estuaries and inland salt flats in arid and semiarid zones [8]. Nowadays, climate-smart agriculture (CSA) practices increasingly use wild salt-tolerant species (halophytes) to restore the grazing capacity of degraded pastures, provide forage for livestock and utilize oilseeds and medicinal and aromatic plants [7,9]. Apart from these applications, halophytes play a significant role in the maintenance of ecosystem functions and sustainability [10,11].

Genetic diversity provides plants with the ability to adapt and survive in changing environments, including soil chemistry variability. The genetic architecture of a population plays a fundamental role in the origin and maintenance of local adaptation [12]. The degree of local adaptation is largely determined by the interaction between selection and gene flow along ecological gradients. Different types of selection can operate under natural conditions: (i) conditionally neutral selection occurs when two alleles do not have an advantage in fitness in one environment but differ in fitness in another environment; (ii) environmentally antagonistic selection, when different alleles are locally adapted to different environments, conferring higher fitness there [12]. ‘Fitness’ is often viewed as the ability to withstand adverse conditions; however, from an evolutionary perspective, fitness is defined as the ability of an individual to spread their genes through offspring. Thus, in plants, fitness depends on the number of seeds that a plant can successfully produce under adverse environmental conditions [13]. A common plant response to environmental stresses is a decrease in fertility, which consists of aborting ovules and/or pollen and redirecting resources from reproductive activity into metabolic reactions for stress tolerance [14]. Plants are reported to control the consumption of maternal resources at several stages of development by regulating the number of flowers, gametophytes, and embryos that develop further [14]. This type of developmental regulation can lead to the favored selection of certain alleles or genotypes, producing genotype–environment associations and/or interactions [15]. The survival rate and adaptation of populations in different and changing environments depend on the genetic diversity of the seed pool. For example, diversity in the genetic composition of seeds allows *Atriplex tatarica* to survive under distinct conditions: heterozygous plants mainly germinate under optimal conditions, and homozygous plants typically germinate under suboptimal conditions [16]. Ecological factors that influence reproduction and seed dispersal are, therefore, particularly important aspects in shaping genetic diversity and population structures. Edaphic conditions, such as soil type, pH, nutrients, moisture, and the depth of soil layers, can significantly affect the level of genetic diversity and local adaptation in plant populations [15,17,18,19]. For example, *Phragmites australis* populations with high genetic diversity have a high tolerance to soil salinity [20]. The high genetic diversity of populations is fundamental to the long-term survival success of a plant species [21].

In unfavorable environments, such as areas of high soil salinity, plants are forced to seek a ‘compromise’ between productivity and adaptation, which depends on genetic diversity at the population level. Within the soil profile, salinity is a dynamic indicator; it changes with depth and according to seasons [5]. In turn, plants have a vertical fine-root distribution, which determines the possibility of acquiring resources along the soil profile, since plants rely mainly on their fine roots to acquire belowground water and nutrient resources [22]. To assess and predict the productivity and adaptation of species under changing conditions, it is necessary to understand how the salinization of different soil layers affects biomass and genetic polymorphism formation. A convenient model species for these purposes is the polymorphic widespread halophyte *Bassia prostrata* (L.) A.J. Scott (*Kochia prostrata* (L.) Schrad.) (subfamily Chenopodiaceae), with a significant variety of morphological, biochemical, and ecological–physiological properties; high genetic polymorphism; and wide ecological plasticity [23,24,25,26,27]. Moreover, the effect of soil conditions on both the level of diversity and genetic structure of *B. prostrata* populations has been shown [28].

The present study aims to investigate the effects of the level and chemistry of salinity within different soil layers (including horizontal and vertical variations in the soil characteristics) on the productivity and genetic diversity of the halophyte *B. prostrata* to clarify the adaptive mechanism it uses to withstand fluctuations of salt accumulation along soil depth profiles.

## 2. Materials and Methods

### 2.1. Study Area

**Figure 1:**
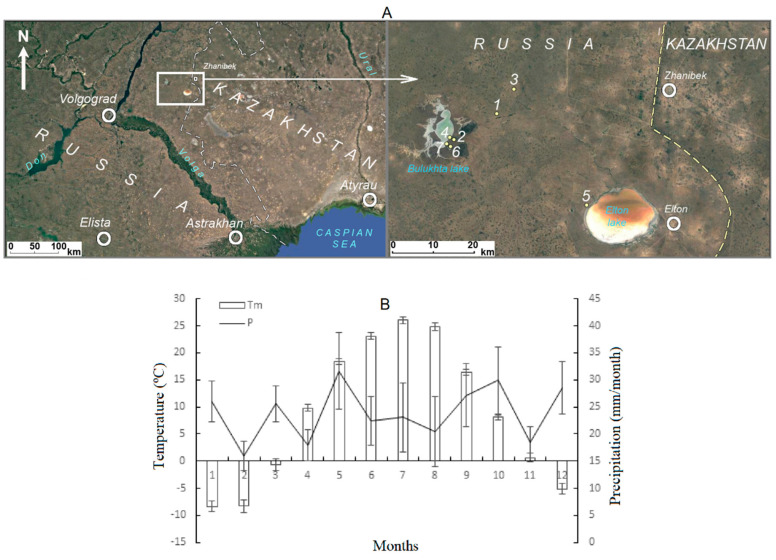
The location of seven populations of *Bassia prostrata* (**A**) and the long-term (2007–2018) average atmospheric temperature and precipitation (**B**) of the northwestern Caspian Lowland. 1–7—numbers of populations (habitats); Tm—temperature; P—precipitation.

Studies were carried out in the northwestern Caspian Lowland (Russia) (Figure 1A). The region is a flat marine accumulative plain, which is characterized by the almost complete absence of surface and subsurface runoff. According to climate parameters (Figure 1B), the region is arid, with an average annual temperature of 8.7 °C and precipitation of 291 mm. In Caspian Lowland plain landscapes, solonetzic complexes are widespread; depressions and other negative relief elements (microdepressions, depressions, estuaries) are characterized by dark-colored chernozem-like or meadow–chestnut soils [29]. Seven typical habitats of *B. prostrata* were selected for the study based on differences in the soil salinity levels (No. 1–7 in Figure 1A). Five of them were located near the salt lakes Bulukhta and Elton at different distances from the coastline. The other two habitats were in the plain part between these lakes (Figure 1A). *B. prostrata* habitats were characterized mainly by solonetzic and/or light chestnut solonetzic soils and desert steppe vegetation.

### 2.2. Plant Sampling

*Bassia prostrata* (L.) A.J. Scott (*Kochia prostrata* (L.) Schrad.) (Chenopodiaceae) is a typical perennial C_4_ halophyte native to arid and semiarid rangelands in Central Eurasia and the Western United States. *B. prostrata* naturally occurs in all kinds of soils, such as saline, sandy, rocky, and poor soils [24,30,31]. *B. prostrata* has a thick, woody root system that can penetrate 3–6.5 m depths and lateral roots stretching 130–160 cm that mine for moisture in the upper (up to 60 cm) soil layers [30,31]. This is the reason for studying the upper soil layers: approximately 0–20 cm, 20–40 cm, and 40–60 cm. Soils, plants, and seeds were sampled in seven typical habitats of *B. prostrata* (No. 1–7 in Figure 1A). The aboveground parts of five plants were harvested in each habitat in the middle of September for biomass analysis. More than 100 seeds from 10 to 15 mother plants from each habitat (population) were collected at the beginning of November and combined to generate a seed pool for population genetic analysis.

### 2.3. Soil Sampling and Analysis

Seven habitats of *B. prostrata* soil pits (Nos. 14, 11, 15, 10, 18, 7, and 6, corresponding to habitats Nos. 1 to 7 in Figure 1A) were excavated. Profiles were examined to depths of 0 to 60 cm. Three soil samples (*n* = 3) were used for the analysis of each soil layer of each habitat. Chemical and physicochemical analyses were performed at the Analytical Laboratory of the V.V. Dokuchaev Soil Science Institute using standard methods [32]. Calcium and magnesium concentrations in water extracts (1:5) were determined with the complexometric titration method; sodium and potassium concentrations were determined with the flame photometry method; the total alkalinity was determined using titration with sulfuric acid (with methyl orange indicator); the concentration of chlorine ions was determined with argentometry (according to Mohr); and the concentration of sulfate ions was determined using titration with BaCl_2_. The content of ions Na^+^, K^+^, Ca^2+^, Mg^2+^, Cl^−^, SO_4_^2−^, and HCO_3_^−^ are presented in cmol(eq)/kg. The sum of salts (S_salts_) represents the sum of the mass fraction of ions from the solid soil residue (%) [6].

### 2.4. Plant Biomass and Water Content

Plant height, fresh (FW) and dry (DW) biomass, and water content (W) were assessed for aboveground parts of *B. prostrata* plants (*n* = 5) from seven habitats. Biomass was estimated for fresh and dry shoots. Plant samples were dried at 80 °C for two days until reaching a constant mass to quantitatively measure the dry shoot matter. The water content in the shoots was calculated according to the following formula:

W = (FW − DW)/DW.(1)

### 2.5. Population Genetic Analysis

Genetic diversity can be studied using neutral markers (based on differences in DNA sequences) and partially selective markers (isozymes), which can reflect changes in environmental conditions [33,34]. In this study, we used isozymes (alternative forms of the enzymes encoded by different alleles of the same gene) to assess the genetic diversity of the populations.

For each population of *B. prostrata*, 50 seeds from the seed pool (more than 100 seeds from 10 to 15 mother plants) were germinated, and all good germinated seeds (*n* = 25–35 per population) were analyzed for genetic polymorphism. Population genetic analysis was performed on embryos using starch gel electrophoresis of the following enzymatic systems: glutamate oxaloacetate transaminase (GOT (AAT), E.C. 2.6.1.1), diaphorase (DIA, E.C. 1.6.99), glutamate dehydrogenase (GDH, E.C. 1.4.1.2), superoxide dismutase (SOD, E.C. 1.15.1.1), glucose-6-phosphate dehydrogenase (G6PD, E.C. 1.1.1.49), 6-phosphogluconate dehydrogenase (6PGD, E.C. 1.1.1.44), malate dehydrogenase (MDH, E.C. 1.1.1.37), and malic enzyme (Me, E.C. 1.1.1.40). The seeds were cleaned of their wings and soaked in water for 12 h and homogenized in 80 μL of Tris-HCl buffer with KCl, MgCl_2_, EDTA, Triton X-100, and PVP. Enzymes were separated in 10% starch gel using two buffer systems. In system 1, the electrode buffer was 160 mM Tris–50 mM citric acid, pH 8.0; the gel buffer was prepared by diluting 10 mL of the electrode buffer with 90 mL H_2_O. In system 2, the electrode buffer was 300 mM boric acid–60 mM NaOH, pH 8.2; the gel buffer was 80 mM Tris–9 mM citric acid, pH 8.7. Electrophoresis was performed at 90 V, 40–50 mA in buffer system 1 or at 210 V, and 70–80 mA in buffer system 2 for 4–6 h at 5 °C. Staining of particular enzymes and genetic interpretation of the results followed standard techniques according to Soltis and Soltis [35] and Spooner et al. [33]. The level of genetic polymorphism was estimated by calculating observed (*H*_o_) and expected (*H*_e_) heterozygosity for each polymorphic loci and by calculating the proportion of polymorphic loci (P_99_) and the average (for all loci) observed (*H*_o_) and expected (*H*_e_) heterozygosity in POPGEN 1.32.

### 2.6. Statistical Analysis

**Figure 2:**
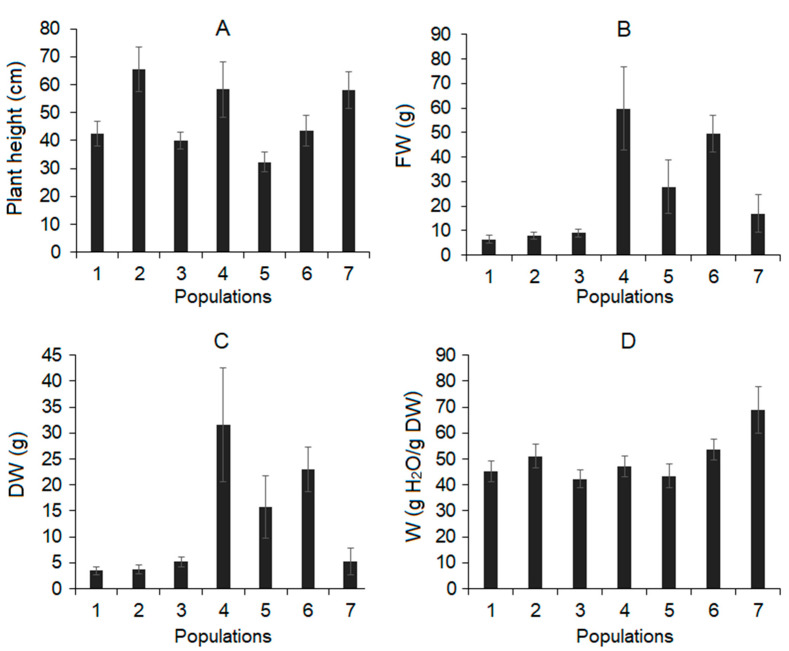
Growth parameters and water content of *Bassia prostrata* plants from seven populations. 1–7—populations; (**A**)—plant height; (**B**)—fresh biomass (FW) aboveground part of plants; (**C**)—dry biomass (DW) aboveground part of plants; (**D**)—water content (W) of plants. Values are means ± standard errors (*n* = 5).

Principal component analysis (PCA) was carried out using R software (version 3.6.1). Table 1 and Figure 2 show the means of the obtained values and their standard errors (*n* = 3 for soil samples and *n* = 5 for plant samples).

## 3. Results

### 3.1. Soil Characteristics

The soils in *B. prostrata* habitats (populations) differed significantly in the degree and vertical changes in salinity chemistry. In each of the seven habitats, the soil salinity the 0–20 cm, 20–40 cm, and 40–60 cm in layers was studied. Two habitats (Nos. 1 and 2) had non-saline or slightly saline soils (Table 1). The soils in the other five habitats (Nos. 3–7) were much more saline: the upper 0–20 cm layers were non-saline or slightly saline (S_salts_ = 0.11–0.25%); the 20–40 cm layers were moderately or highly saline (S_salts_ = 0.5–1.17%); and the 40–60 cm layers were highly or extremely saline (S_salts_ = 1.35–2.57%) (Table 1). In all habitats, except for No. 4, Na^+^ was the dominant cation: 0.03–2.27 cmol(eq)/kg, 0–20 cm layer; 0.32–8.95 cmol(eq)/kg, 20–40 cm layer; and 3.15–23.25 cmol(eq)/kg, 40–60 cm layer. The Ca^2+^ ion predominated in habitat No. 4. In other soils, Ca^2+^ and Mg^2+^ contents also significantly contributed to salinity at 0.28–12.9 and 0.26–8.32 cmol(eq)/kg, respectively. Chlorides and sulfates were the dominant anions (Table 1).

**Table 1:** Contents of anions and cations in soils of the seven *Bassia prostrata* habitats in the northwestern Caspian Lowland.

Habitats, No	Soil Layers	Anions, cmol(eq)/kg			Cations, cmol(eq)/kg				Ssalt, %	Salinity Level
		HCO^3−^	Cl^−^	SO_4_^2−^	Ca^2+^	Mg^2+^	Na^+^	K^+^		
1	0–20	1.04 ± 0.08	0.26 ± 0.02	0.78 ± 0.04	0.78 ± 0.02	0.52 ± 0.04	0.56 ± 0.01	0.22 ± 0.04	0.15	non-saline
	20–40	1.25 ± 0.11	0.17 ± 0.01	0.52 ± 0.03	0.52 ± 0.01	0.26 ± 0.03	1.45 ± 0.12	0.02 ± 0.01	0.16	non-saline
	40–60	2.18 ± 0.17	0.26 ± 0.03	0.52 ± 0.03	0.52 ± 0.02	0.78 ± 0.08	1.74 ± 019	0.03 ± 0.01	0.23	slight
2	0–20	0.10 ± 0.02	0.17 ± 0.01	0.52 ± 0.03	0.26 ± 0.01	0.26 ± 0.01	0.24 ± 0.01	0.03 ± 0.01	0.05	non-saline
	20–40	0.31 ± 0.02	0.34 ± 0.04	0.26 ± 0.01	0.26 ± 0.02	0.26 ± 0.01	0.32 ± 0.04	0.07 ± 0.01	0.06	non-saline
	40–60	0.94 ± 0.13	1.89 ± 0.14	1.82 ± 0.23	0.78 ± 0.66	0.78 ± 0.32	3.15 ± 0.07	0.04 ± 0.01	0.31	slight
3	0–20	1.35 ± 0.08	0.34 ± 0.07	0.52 ± 0.01	0.52 ± 0.02	0.26 ± 0.01	1.31 ± 0.04	0.12 ± 0.01	0.17	slight
	20–40	1.77 ± 0.21	5.41 ± 0.43	1.04 ± 0.08	0.52 ± 0.01	0.78 ± 0.03	7.01 ± 0.16	0.01 ± 0.01	0.50	moderate
	40–60	0.52 ± 0.02	10.22 ± 0.93	18.98 ± 0.21	9.88 ± 0.10	8.32 ± 0.72	11.48 ± 0.81	0.04 ± 0.01	1.87	extreme
4	0–20	0.94 ± 0.10	0.26 ± 0.03	1.56 ± 0.33	2.08 ± 0.50	0.26 ± 0.09	0.14 ± 0.07	0.27 ± 0.05	0.20	slight
	20–40	0.57 ± 0.04	0.17 ± 0.01	15.60 ± 0.42	12.09 ± 0.74	3.12 ± 0.41	1.84 ± 0.09	0.15 ± 0.01	1.11	moderate
	40–60	0.52 ± 0.03	0.69 ± 0.08	18.72 ± 1.53	9.88 ± 0.51	3.64 ± 0.33	6.27 ± 0.71	0.14 ± 0.02	1.35	high
5	0–20	0.73 ± 0.17	0.49 ± 0.0.1	0.35 ± 0.07	0.43 ± 0.08	0.43 ± 0.11	0.74 ± 0.26	0.06 ± 0.02	0.11	non-saline
	20–40	1.25 ± 0.76	7.90 ± 0.06	2.60 ± 0.32	1.04 ± 0.09	0.52 ± 0.07	10.28 ± 0.93	0.01 ± 0.01	0.75	moderate
	40–60	0.31 ± 0.02	7.39 ± 0.07	23.92 ± 1.30	10.14 ± 0.12	6.76 ± 0.54	14.77 ± 1.10	0.05 ± 0.01	2.06	extreme
6	0–20	0.78 ± 0.13	0.39 ± 0.04	0.91 ± 0.11	0.52 ± 0.01	0.52 ± 0.01	0.78 ± 0.07	0.26 ± 0.09	0.15	non-saline
	20–40	1.77 ± 0.17	3.18 ± 1.05	4.03 ± 0.98	1.04 ± 0.21	0.39 ± 0.11	7.50 ± 1.94	0.04 ± 0.01	0.76	moderate
	40–60	0.42 ± 0.03	7.65 ± 1.11	22.36 ± 1.74	11.70 ± 1.03	8.06 ± 0.92	10.67 ± 1.05	0.10 ± 0.03	1.95	extreme
7	0–20	1.51 ± 0.04	1.42 ± 0.74	1.69 ± 0.32	0.52 ± 0.01	0.39 ± 0.11	3.51 ± 1.01	0.20 ± 0.11	0.25	slight
	20–40	0.73 ± 0.04	9.02 ± 1.08	8.58 ± 0.91	3.12 ± 0.07	2.08 ± 0.34	13.14 ± 1.11	0.09 ± 0.01	1.17	high
	40–60	0.62 ± 0.03	16.06 ± 1.77	23.66 ± 1.73	10.66 ± 0.85	6.50 ± 0.77	23.25 ± 2.76	0.04 ± 0.01	2.57	extreme

Ssalt—the sum of salts represents the sum of the mass fraction of ions from the solid soil residue (%). Values are means ± standard errors (*n* = 3).

### 3.2. Plant Growth and Water Content

*B. prostrata* plants varied significantly in growth parameters between populations (Figure 2). The greatest plant heights were in populations Nos. 2, 4, and 7 (Figure 2A), while the highest fresh (FW) and dry (DW) biomass aboveground parts of plants were observed in populations No. 4 and No. 6 (Figure 2B,C). At the same time, the water content (W) in the plants was the highest in population No. 7 (Figure 2D).

### 3.3. Population Genetic Diversity

**Figure 3:**
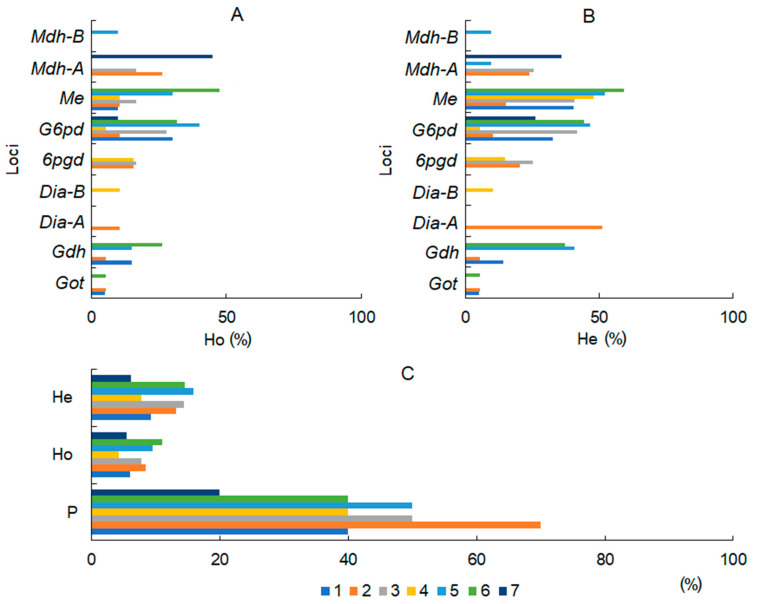
Genetic polymorphism in seven populations of *Bassia prostrata.* (**A**)—observed heterozygosity (*H*_o_) of polymorphic loci; (**B**)—expected heterozygosity (*H*_e_) of polymorphic loci; (**C**)—polymorphic loci proportion of population (*P*), average (for all loci) observed heterozygosity (*H*_o_), and average (for all loci) heterozygosity (*H*_e_) of seven populations of *Bassia prostrata*.

An analysis of eight enzyme systems in seven *B. prostrata* populations revealed ten loci; one of them (*Sod*) was monomorphic in all populations. The other nine loci were polymorphic: *G6pd* in all populations; *Me* in six populations; *Gdh* in four populations; *Got, 6pgd*, and *Mdh-A* in 3 populations; and *Dia-A, Dia-B*, and *Mdh-B* in one population. Values of observed heterozygosity (*H*_o_) varied from 5 to 47% among polymorphic loci and populations (Figure 3A), whereas expected heterozygosity (*H*_e_) varied from 5% to 59% (Figure 3B). The average (for all loci) observed heterozygosity varied from 5.5% to 11.1%, and expected heterozygosity varied from 6.2% to 15.9% in populations of *B. prostrata* (Figure 3C). The polymorphic loci proportion (*P*) among the populations was 20–70% (Figure 3C). On average, populations Nos. 2, 3, 5, and 6 were more polymorphic than populations Nos. 1, 4, and 7 (Figure 3).

### 3.4. Plant–Soil Interaction

**Figure 4:**
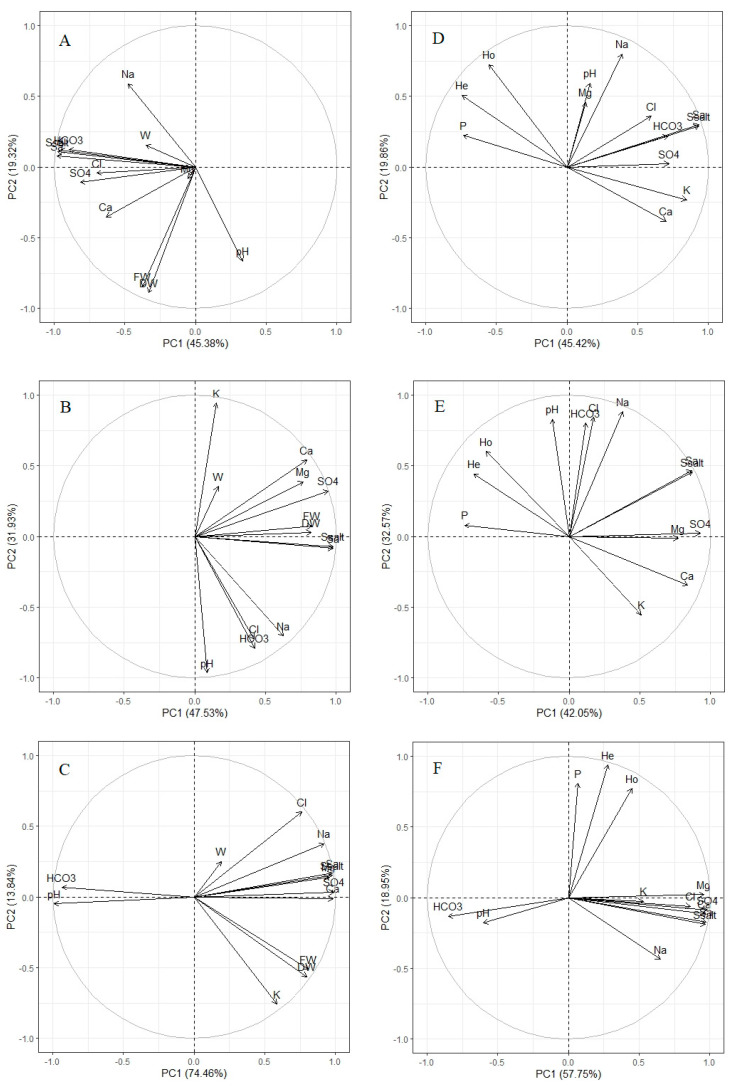
Principle component analysis (PCA) of growth (**A**–**C**), genetic diversity (**D**–**F**) parameters of *Bassia prostrata*, and salinity of 0–20 cm (**A**,**D**), 20–40 cm (**B**,**E**), and 40–60 cm (**C**,**F**) soil layers. K^+^, Na^+^, Ca^2+^, Mg^2+^, Cl^−^, SO_4_^2−^, HCO_3_^−^—ions content in soil; Ss—sum of salts; Sa—sum of anions in soil; FW—fresh plant biomass; DW—dry plant biomass; W—water content in leaves; *P*—proportion of polymorphic loci; *H*_o_—observed heterozygosity; and *H*_e_—expected heterozygosity of *B. prostrata*.

Principal component analysis (PCA) did not reveal significant correlations between *B. prostrata* fresh and dry biomass and soil properties in 0–20 cm soil layers (Figure 4A). There were significant positive correlations between *B. prostrata* fresh and dry biomass and the sum of salts and the sum of the contents of anions Ca^2+^, Mg^2+^, and SO_4_^2−^ in the 20–40 cm and, to a lesser degree, 40–60 cm soil layers (Figure 4B), as well as with K^+^ content in 40–60 cm layers (Figure 4C).

PCA revealed the negative dependencies of genetic polymorphism parameters (*P*, *H*_e_, and *H*_o_) on K^+^, Ca^2+^, and sulfate ions contents and, to a lesser degree, on the sum of salts and the sum of anions in the 0–20 cm soil layers (Figure 4D). In addition, a negative correlation was observed between *P, H*_e_, and *H*_o_ from one side and Mg^2+^, K^+^, Ca^2+^, and SO_4_^2−^ contents in 20–40 cm soil layers from the other side (Figure 4E). There were no correlations between genetic polymorphism parameters and soil properties in the 40–60 cm layers (Figure 4F).

## 4. Discussion

The habitats of *Bassia prostrata* in this study were characterized by significant diversity in the degree and chemistry of soil salinity; high salinity occurred at different soil depths (Table 1). *B. prostrata* has wide edaphic plasticity and can grow on various soil genesis, e.g., chestnut, light-chestnut alkaline soils, and solonetz, as well as on soil-forming rocks of different compositions, from light sandy to heavy loamy, stony, and gypsum [30,36].

Our results revealed differences in correlations between *B. prostrata* aboveground biomass accumulation and seed genetic polymorphism and the chemistry and degree of salinity of different soil layers. The genetic diversity level was affected by the salinity degree and the chemistry of the uppermost soil layers (0–20 cm, 20–40 cm), and biomass accumulation was mainly affected by the salinity of the 20–40 cm and 40–60 cm soil layers. Such differences may be associated with different seasons of aboveground biomass and seed pool formation. *B. prostrata* biomass accumulation (before flowering) occurs mainly in the summer, the hottest and driest season: 23–26 °C, 40–43% humidity, and 65.7 mm precipitation (Figure 1B). In the summer, the drying of the uppermost soil layers can be observed, and plants receive water and dissolved salt ions from lower soil layers, affecting biomass formation. Our study showed a positive dependence of *B. prostrata* productivity on the degree of salinity in 20–40 cm soil layers (Figure 4B). *B. prostrata*, as a halophyte, requires a certain amount of salt in the substrate for optimal growth [37] and has high productivity in soils with 20 dS/m (EC) salinity [31]. The content of the main plant nutrient K^+^ in seven soil habitats decreased from the upper to lower layers, whereas the Na^+^ concentration increased (means of K^+^/Na^+^ were 0.45 and 0.01 in the 0–20 cm and 40–60 cm soil layers, respectively; Table 1). Despite the fact that plants growing in saline habitats have acquired mechanisms that allow for selective uptake of K^+^ when Na^+^ dominates in the substrate [37], in *B. prostrata* plants, K^+^ content in tissues decreased when Na^+^ exceeded 100–200 mM NaCl [38]. Thus, the selective absorption of K^+^ from the 40–60 cm soil layer under conditions of increased competition with Na^+^ affects *B. prostrata* biomass accumulation in natural habitats (Figure 4C).

Ca^2+^ and Mg^2+^ ions are also essential mineral nutrients. Ca^2+^ is a universal signal in all eukaryotic cells and participates in many other cellular processes, for example, in the maintenance of cell membrane integrity, cation–anion balance, and osmoregulation [39,40]. Mg^2+^ is an activator of more than 300 enzymes, in particular, photosynthetic and respiratory ones, which are also needed for DNA and RNA synthesis [41,42]. It is well known that Ca^2+^ plays a protective role in a plant’s response to salinity. Much less is known about the role of Mg^2+^ in the salt tolerance of plants [39]. However, it was shown that low concentrations of mixed salts with CaCl_2_, and MgSO_4_ are necessary for the successful seed germination of *B. prostrata* [43]. Our study showed that Ca^2+^ and Mg^2+^ contents contributed significantly to soil salinity in *B.prostrata* habitats (Table 1). Positive correlations between biomass accumulation and Ca^2+^ and Mg^2+^ contents in the 20–40 cm soil layer (Figure 4B) indicate their necessity for *B. prostrata* growth. The influence of magnesium in this soil layer can be associated with the optimal K^+^/Mg^2+^ ratio. The K^+^/Mg^2+^ ratio for soils and plant tissues is critical to maintaining optimal plant nutrition and, hence, plant productivity [42]. The K^+^/Mg^2+^ ratio (0.09 ± 0.03) in the 20–40 cm soil layer in *B. prostrata* habitats was less than that of the 0–20 cm soil layer (0.56 ± 0.17) but higher than that of the 40–60 cm soil layer (0.02 ± 0.01).

*B. prostrata* seeds are formed in autumn, during a cooler and rainier period (1–16 °C, 49–81% humidity, 77.1 mm precipitation; Figure 1B) when the upper soil layers are moist and plants receive water and dissolved salt ions from them. At the same time, the need for water decreases due to lower air temperatures and higher humidity. Therefore, the formation of seed genetic diversity in *B. prostrata*, upon which the future stability of populations in changing environments depends [12,44,45], is affected by the salinity level and ionic composition of the 0–40 cm soil layers. In heterogeneous environments, the processes of gene flow, mutation, and sexual reproduction generate local genetic variation, providing material for local adaptation [45]. The influence of soil factors such as soil type, pH, moisture, and soil layer depth on population genetic diversity has been demonstrated in different plant species [15,17,18,19]. A nine-year experiment on the influence of soil moisture and nitrogen, phosphorus, and potassium content in soil on allozyme frequency revealed an allele–habitat association in *Festuca ovina* [15]. It was found that in natural populations the *Pgi*-2-2 allele is significantly associated with soil moisture and is affected by nutrient/water treatments [15]. Negative correlations between *B. prostrata* genetic diversity with inorganic ion content (except for Na^+^ and Cl^−^) and the sum of salts in the 0–40 cm soil layers (Figure 4D,E) indicate selection in favor of homozygotes. Since isozymes (allozymes) were also used in our study, a question arises regarding the functional significance of enzymes under selection. Loci *G6pd* and *Me* were the most polymorphic among the *B. prostrata* populations (Figure 3A). They encode the enzymes glucose-6-phosphate dehydrogenase (G6PD) and malik-enzyme (NADP-Me), respectively, which are associated with the regulatory nodes of dark respiration and photosynthesis. G6PD is a key enzyme in the alternative apotomous oxidative pentose phosphate pathway (OPPP), whose role is enhanced under stress [46]. Malik-enzyme is involved in photosynthesis and is especially active in C_4_ species, and it plays a vital role in the tolerance to salt stress [47]. The adaptive–compensatory reactions of plants under stress are always associated with additional energy costs, which leads to a change in the balance between photosynthesis and respiration [46]. Any shifts in this balance are reflected in the total plant productivity. Selection leads to local adaptation, and the strength of local adaptation depends on the strength of selection. Strong selection leads to strong local adaptation, which is significantly affected by landscape heterogeneity [48]. The negative influence of Ca^2+^, Mg^2+^, and SO_4_^2−^ contents in the 0–40 cm soil layer on heterozygosity indicates the formation of the local adaptation of *B. prostrata* to magnesium–calcium sulfate soil salinity. The detected level of sodium chloride salinity did not negatively impact seed genetic polymorphism (Figure 4D,E). This is probably due to the necessity of these ions in maintaining water balance in the aboveground organs of *B. prostrata* (Figure 4C).

## 5. Conclusions

Our study demonstrates that in natural habitats the productivity and seed genetic polymorphism of halophytes may be affected by the salinity of different soil layers. These differential plant responses to vertically heterogeneous soil salinity could be attributed to seasonal variables during biomass accumulation (summer) and seed formation (autumn). An excess of some ions in the uppermost soil layers can lead to increased local adaptation to a certain type of salinity and the appearance of genotype-environment associations. Genotype–environment association analyses may allow us to develop adaptive measures for natural resource management, pasture improvement, and the phytoremediation and restoration of lands with different salinity chemistries.

## Data Availability

The data are available from the authors upon request.

## References

[1] Huang J., Ji M., Xie Y., Wang S., He Y., Ran J. (2016). Global semi-arid climate change over last 60 years. Clim. Dyn..

[2] Acosta-Motos J.R., Ortuño M.F., Bernal-Vicente A., Diaz-Vivancos P., Sanchez-Blanco M.J., Hernandez J.A. (2017). Plant responses to salt stress: Adaptive mechanisms. Agronomy.

[3] Dantas B.F., Moura M.S.B., Pelacani C.R., Angelotti F., Taura T.A., Oliveira G.M., Bispo J.S., Matias J.R., Silva F.F.S., Pritchard H.W. (2020). Rainfall, not soil temperature, will limit the seed germination of dry forest species with climate change. Oecologia.

[4] Welegedara N.P.Y., Grant R.F., Quideau S.A., Landhäusser S.M., Merlin M., Lloret E. (2020). Modelling plant water relations and net primary productivity as affected by reclamation cover depth in reclaimed forestlands of northern Alberta. Plant Soil.

[5] Kakeh J., Gorji M., Mohammadi M.H., Asadi H., Khormali F., Sohrabi M., Cerdà A. (2020). Biological soil crusts determine soil properties and salt dynamics under arid climatic condition in Qara Qir, Iran. Sci. Total Environ..

[6] Pankova E., Vorob’eva L., Gadjiev I., Gorohova I.N., Elizarova T.N., Koroluk T.V., Lopatovskaya O.G., Novikova A.F., Reshetov G.G., Skripnikova M.I. (2006). Saline Soils in Russia.

[7] Flowers T., Galal H., Bromham L. (2010). Evolution of halophytes: Multiple origins of salt tolerance in land plants. Funct. Plant Biol..

[8] Yamanaka N., Toderich K. (2020). Photobooks of Drylands Vol.4 Salinization in Drylands.

[9] Nikalje G.C., Yadav K., Penna S., Hasanuzzaman M., Nahar K., Öztürk M. (2019). Halophyte Responses and Tolerance to Abiotic Stresses. Ecophysiology, Abiotic Stress Responses and Utilization of Halophytes.

[10] Flowers T.J., Colmer T.D. (2015). Plant salt tolerance: Adaptation in halophytes. Ann. Bot..

[11] Ventura Y., Eshel A., Pasternak D., Sagi M. (2015). The development of halophyte-based agriculture: Past and present. Ann. Bot..

[12] Tigano A., Friesen V.L. (2016). Genomics of local adaptation with gene flow. Mol. Ecol..

[13] Thalmann M., Santelia D. (2017). Starch as a determinant of plant fitness under abiotic stress. New Phytol..

[14] Sun K., Hunt K., Hauser B.A. (2004). Ovule abortion in Arabidopsis triggered by stress. Plant Physiol..

[15] Prentice H., Lonn M., Lager H., Rosen E., van der Maarel E. (2000). Changes in allozyme frequencies in *Festuca ovina* populations after a 9-year nutrient/water experiment. J. Ecol..

[16] Mandak B., Bimova K., Plackova I. (2006). Genetic structure of experimental populations and reproductive fitness in a heterocarpic plant *Atriplex tatarica* (Chenopodiaceae). Am. J. Bot..

[17] Nevo E., Brown A., Zohary D., Storch N., Beiles A. (1981). Microgeographic edaphic differentiation an allozyme polymorphysms of wild barley (*Hordeum spontaneum*, Poaceae). Plant Syst. Evol..

[18] Nevo E., Krugman T., Beiles A. (1994). Edaphic natural selection of allozyme polymorphisms in *Aegilops peregrina* at a Galilee microsite in Israel. Heredity.

[19] Prentice H., Lonn M., Lefkovitch L., Runyeon H. (1995). Associations between allele frequencies in *Festuca ovina* and habitat variation in the alvar grass-lands on the Baltic island of Oland. J. Ecol..

[20] Sun X.-S., Chen Y.-H., Zhuo N., Cui Y., Luo F.-L., Zhang M.-X. (2021). Effects of salinity and concomitant species on growth of *Phragmites australis* populations at different levels of genetic diversity. Sci. Total Environ..

[21] Aavik T., Helm A. (2018). Restoration of plant species and genetic diversity depends on landscape-scale. Restor. Ecol..

[22] Song X., Gao X., Wu P., Zhao X., Zhang W., Zou Y., Siddique K.H.M. (2020). Drought responses of profile plant-available water and fine-root distributions in apple (*Malus pumila* Mill.) orchards in a loessial, semiarid, hilly area of China. Sci. Total Environ..

[23] Shuyskaya E.V., Toderich K.N., Voinitska-Poltorak A. (2001). Genetic variation of *Kochia prostrata* (L.) Schrad. in the arid zone of Uzbekistan. Probl. Osvoeniya Pustyn..

[24] Gintzburger G., Toderich K.N., Mardonov B.K., Makhmudov M.M. (2003). Rangelands of the Arid and Semi-Arid Zones in Uzbekistan.

[25] Akhzari D., Sepehry A., Pessarakli M., Barani H. (2012). Studying the effects of salinity stress on the growth of various halophytic plant species (*Agropyron elongatum*, *Kochia prostrata* and *Puccinellia distans*). World Appl. Sci. J..

[26] Toderich K.N., Shuyskaya E.V., Taha F.K., Matsuo N., Ismail S., Aralova D.B., Radjabov T.F., Shahid S., Abdelfattah M., Taha F. (2013). Integrating Agroforestry and Pastures for Soil Salinity Management in Dryland Ecosystems in Aral Sea Basin. Developments in Soil Salinity Assessment and Reclamation.

[27] Shuyskaya E., Rakhmankulova Z., Prokofieva M., Saidova L., Toderich K., Voronin P. (2022). Intensity and duration of salinity required to form adaptive response in C_4_ halophyte *Kochia prostrata* (L.) Shrad. Front. Plant Sci..

[28] Shuyskaya E.V., Nukhimovskaya Y.D., Lebedeva M.P., Churilina A.E., Kolesnikov A.V. (2020). Effect of soil conditions on the level of genetic diversity in the xerohalophyte *Kochia prostrata* (L.) Schrad. (Chenopodiaceae). Russ. J. Ecol..

[29] Konyushkova M., Kozlov D. (2011). Automated analysis of the distribution of dark-colored chernozemlike soils in the Northern Caspian region based on satellite imaging data: The example of the Dzhanybek station. Arid Ecosyst..

[30] Balyan G. (1972). Kochia Prostrata in Kyrgyzstan.

[31] Waldron B., Eun J., ZoBell D., Olson K. (2010). Forage kochia (*Kochia prostrata*) for fall and winter grazing. Small Rumin. Res..

[32] Vorob’eva L.A. (1998). Chemical Analysis of Soils.

[33] Spooner D., van Treuren R., de Vicente M. (2005). Molecular Markers for Genebank Management.

[34] Marden J.H. (2013). Nature’s inordinate fondness for metabolic enzymes: Why metabolic enzyme loci are so frequently targets of selection. Mol. Ecol..

[35] Soltis D., Soltis P. (1990). Isozymes in Plant Biology.

[36] Harrison R.D., Chatterton N.J., Waldron B.L., Davenport B.W., Palazzo A.J., Horton W.H., Asay K.H. (2000). Forage Kochia: Its Compatibility and Potential Aggressiveness on Intermountain Rangelands.

[37] Flowers T.J., Colmer T.D. (2008). Salinity tolerance in halophytes. New Phytol..

[38] Karimi G., Ghorbanli M., Heidari H., Khavari Nejad R., Assareh M. (2005). The effects of NaCl on growth, water relations, osmolytes and ion content in Kochia Prostrata. Biol. Plant..

[39] Grigore M., Boscaiu M., Llinares J., Vocente O. (2012). Mitigation of salt stress-induced inhibition of *Plantago crassifolia* reproductive development by supplemental calcium or magnesium. Not. Bot. Horti Agrobot. Cluj-Napoca.

[40] Tian W., Wang C., Gao Q., Li L., Luan S. (2020). Calcium spikes, waves and oscillations in plant development and biotic interactions. Nat. Plants.

[41] Shaul O. (2002). Magnesium transport and function in plants: The tip of iceberg. Biometals.

[42] Xie K., Cakmak I., Wang S., Zhang F., Guo S. (2021). Synergistic and antagonistic interactions between potassium and magnesium in higher plants. Crop. J..

[43] Orlovsky N.S., Japakova U.N., Shulgina I., Volis S. (2011). Comparative study of seed germination and growth of *Kochia prostrata* and *Kochia scoparia* (Chenopodiaceae) under salinity. J. Arid Environ..

[44] Loveless M.D., Hamrick J.L. (1984). Ecological determinants of genetic structure in plant populations. Ann. Rev. Syst..

[45] North A., Pennanen J., Ovaskainen O., Laine A.-L. (2010). Local adaptation in a changing world: The roles of gene-flow, mutation, and sexual reproduction. Evolution.

[46] Rakhmankulova Z.F. (2022). Plant respiration and global climatic changes. Russ. J. Plant Physiol..

[47] Kandoi D., Tripathy B.C. (2022). Overexpression of chloroplastic *Zea mays* NADP-malic enzyme (ZmNADP-ME) confers tolerance to salt stress in *Arabidopsis thaliana*. Photosynth. Res..

[48] Forester B.R., Jones M.R., Joost S., Landguth E.L., Lasky J.R. (2016). Detecting spatial genetic signatures of local adaptation in heterogeneous landscapes. Mol. Ecol..

